# Damage Accumulation in Cyclically-Loaded Glass-Ceramic Matrix Composites Monitored by Acoustic Emission

**DOI:** 10.1155/2013/869467

**Published:** 2013-11-28

**Authors:** D. G. Aggelis, K. G. Dassios, E. Z. Kordatos, T. E. Matikas

**Affiliations:** ^1^Department of Mechanics of Materials and Constructions, Free University of Brussels, Pleinlaan 2, 1050 Brussels, Belgium; ^2^Department of Materials Science and Engineering, University of Ioannina, 45110 Ioannina, Greece

## Abstract

Barium osumilite (BMAS) ceramic matrix composites reinforced with SiC-Tyranno fibers are tested in a cyclic loading protocol. Broadband acoustic emission (AE) sensors are used for monitoring the occurrence of different possible damage mechanisms. Improved use of AE indices is proposed by excluding low-severity signals based on waveform parameters, rather than only threshold criteria. The application of such improvements enhances the accuracy of the indices as accumulated damage descriptors. RA-value, duration, and signal energy follow the extension cycles indicating moments of maximum or minimum strain, while the frequency content of the AE signals proves very sensitive to the pull-out mechanism.

## 1. Introduction

**Figure 1:**
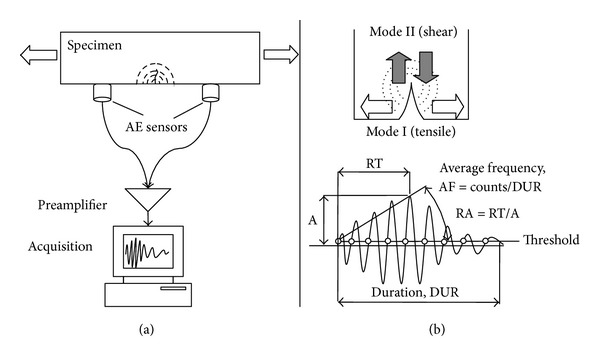
(a) Typical AE application in laboratory and (b) typical AE waveform and fracture modes.

The importance of monitoring the structural safety of structures is imperative. Early assessment of material conditions before large-scale fracture emerges helps not only to prevent destructive failure but also to manage the structures safely and economically. Regular observation and study of deterioration signs can lead to the proper repair action and results in the extension of the useful life-span of the structural component or its replacement if necessary. Nondestructive methods capable of monitoring the structural integrity of the structures in an efficient and economical way are highly sought for. One of the methods used for real time nondestructive monitoring is Acoustic Emission (AE). It is based on the fact that any fracture incident inside a material releases energy. Similarly to seismic activity, though in a shorter scale, this energy propagates in the form of elastic waves and the transient response of the surface is recorded by the use of appropriate sensors [1]; see Figure 1(a). The sensors are usually piezoelectric and transform the energy of the transient elastic wave to an electric waveform which is digitized and stored for further analysis. AE supplies information about the accumulated activity which is connected to the density of active cracks and the sustained load [1–4]. Additionally, the time delay between acquisition of the signals at the different positions leads to calculation of the location of the crack [5, 6]. In cyclic loading histories (loading or thermal) quantitative indices have been applied using the activity during unloading in comparison to the loading stage of the material such as the “calm” ratio [7–9]. Furthermore, related to the well-known “Kaiser effect” [10], the load under which AE is acquired compared to the prior maximum load (felicity ratio) is also a sensitive indicator of damage condition [11, 12]. However, the above mentioned indices employ the total AE activity, even signals of limited importance, while in the present case specific procedures are proposed in order to exclude the less significant signals and improve the clarity of the outcome. Apart from indices based on the onset or the number of emissions, other parameters are based on the qualitative waveform characteristics. The transient elastic waves excited at the tip of the crack contain information on the motion of the crack sides. The amount of the recorded acoustic energy depends on the movement at the crack tip during crack propagation, being, therefore, indicative of the moment of crack initiation, the severity of active cracking, and the extent of damage [13–15] Analysis of AE indices enlightens the characterization of the cracking mode. The importance of this information lies in the fact that usually in structural materials shear phenomena like debonding or delaminations follow tensile ones like matrix cracking. Therefore, characterization of the cracking mode apart from revealing the damage stage of the material also allows conclusions on the remaining life, acting as a warning against final failure. 

Figure 1(b) shows two basic cracking modes and a typical AE waveform. One of the most important waveform characteristics is peak amplitude, A, measured in Volts or dB and being normally correlated with the intensity of the cracking incident. The duration (DUR) and the rise time (RT) of the signal are similarly important. DUR is defined as the time between the first and the last threshold crossing. RT is defined by the delay between the onset of the signal and the time of the peak amplitude. A parameter that has extensively been used for the fracture mode characterization depends on the shape of the initial part of the waveform. The ratio of RT over A, namely, “RA” value is recently employed in structural materials for crack characterization [16, 17]. The average frequency, AF, is another essential parameter defined by the number of threshold crossings over the duration of the signal and is measured in kHz. Initiation frequency, I.F. is the number of threshold crossings during RT over RT (similar to AF of the first part of the waveform). It is known that the frequency content of the waveform is strongly affected by the crack type [16–18]. Additionally, AE energy (ENE) expresses the area under the rectified waveform envelope. Similarly to the amplitude, it expresses the severity of cracking since generation of a large crack should emit larger amount of energy than a small crack. Energy related parameters have been studied in relation to the fracture toughness of ceramics [19] and as predictors of rupture [20]. The direct relation between the micromotion of the crack sides and the emitted waveform is a subject needing extensive discussion. However, it can be mentioned that when shear cracking mode is active, although both bulk elastic wave types (longitudinal and shear) are emitted by the tip of the crack, most of the energy goes into the formation of shear waves. These results are in longer waveforms since shear waves are slower and arrive later than the fast longitudinal ones tending to elongate the acquired waveform. On the other hand, tensile mode of cracking, results in most of the energy being emitted as longitudinal waves and therefore the substantial part arrives early within the waveform. This means that in general, when tensile mode is dominant, waveforms with short RT and low RA are recorded, while when the mode shifts to shear, RA starts to increase among other parameters [16].

In the present study, results of fracture tests in ceramic matrix composites reinforced with SiC fibers are presented and analyzed. The composite was used as a model material since its fracture process includes different mechanisms [21]. Low load induces reopening of thermal microcracks and tensile matrix cracking of the weak off-axis layers. Higher load induces shear phenomena like fiber/matrix debonding, bundle sliding, and fiber pull-out. Compression load has been related to delaminations and fiber bundle buckling [22, 23] while cracking starts at stress levels below one half of the strength [24]. AE is studied in relation to the different damage mechanisms in order to check their fingerprint aiming at real time structural health monitoring. Some indices have been used in a pattern recognition approach for damage characterization in CMCs under fatigue [25]. AE acquisition rate and amplitude have been studied in relation to microcracking of ceramics due to stress and thermal loading [6, 14, 15, 26, 27]. AE has been related to residual stresses [9] and frequency parameters have also been used in order to characterize the occurrence of microdamage in zirconia-based ceramics [28], while waveform parameters like amplitude, threshold crossings and root mean square of the AE signals have been related to the force and velocity of grooving process in ceramic composites [15, 29]. Results show that AE indices under proper study lead to characterization of the current state of structural condition, being sensitive to the load and the active damage mechanisms.

## 2. Experimental Details

### 2.1. Materials and Testing

Cross-ply laminated plates, 3 mm in thickness, were processed by Harwell Ltd, United Kingdom. The glass-ceramic matrix consisted of barium, magnesium, alumina, and silicate (BMAS, barium osumilite). The fibers, silicon carbide grade “Tyranno,” were provided by UBE Industries Ltd., Japan. The composite was processed by hot-pressing pre-preg sheets of desized fibers wet by the glass slurry, in a graphite die at a temperature of 1,200°C. During hot-pressing, a carbon-rich layer is formed at the fibers' surface due to the reaction of SiC with the oxides in the matrix [30, 31]. This layer provides a weak fibre/matrix interface that is responsible for the development of important energy dissipation mechanisms during loading, such as interfacial debonding, fiber sliding, fiber bridging, and pull-out [32].

The double-edge notch (DEN) specimen geometry was selected with variable notch-to-width ratios in order to investigate the effect of notches on the mechanical response of the material as well as to confine fracture within a monitored region. Specimens of dimensions *l* × *w* × *t* = 105 × 12 × 3 mm were cut from the plates in a CNC vertical machining center equipped with a diamond wafering blade. Care was taken so that fiber orientation in the external plies was parallel to the axial direction. Notch-to-width ratios of 0.2 (type A) and 0.35 (B) were used. In order to establish the baseline performance of the unnotched material, specimens of rectangular cross-section were also prepared (C) in the following analysis. For each geometry configuration, sets of three specimens were prepared.

Tensile loading under a constant crosshead displacement rate of 0.2 mm/min was performed on an Instron 8800 servohydraulic test system (Illinois Tool Works, Glenview, IL, USA) equipped with a 100 kN load cell and hydraulic clamping grips. The calculated strain rate within the effective gauge length was 4.0 × 10^−3^ min^−1^. The net gauge length between the grips was approximately 50% of the whole specimen length, 50 mm. Specimens were clamped at 4 MPa without end tabs. A clip-on axial extensometer with knife-edge mounting legs was used to monitor strain within the central 25 mm of the specimen. The loading-unloading cycles that were included during testing provided an instantaneous damage probe of the material. Cycle generation started at 10^−3^ strain with a step of 1.5 × 10^−3^. Upon unloading, the material was allowed to relax completely (zero load). 

### 2.2. AE Monitoring

**Figure 2:**
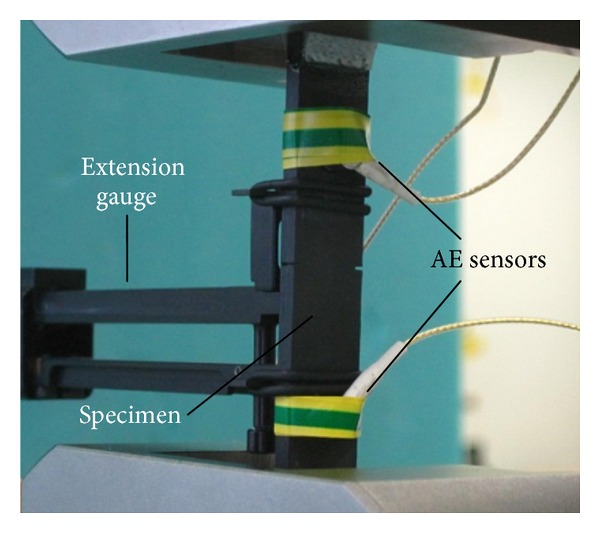
Snapshot during experiment.

For the AE monitoring, two wide band AE sensors (Pico, PAC) were attached on the same side of the specimen (Figure 2) by the use of electric-mounting tape. Silicon grease was applied between the sensors and the specimen to provide acoustic coupling. The specific sensors are sensitive to frequencies from 50 kHz up to approximately 800 kHz, with maximum sensitivity at 450 kHz. Therefore, they are suitable to record wide range of different sources. The distance between the two receivers was set nominally at 40 mm, while after placing, the exact distance was measured. The signals were amplified by 40 dB and in order to avoid ambient noise the threshold was set to 45 dB. The signals were recorded on a two-channel PCI-2 monitoring board of PAC with a sampling rate of 5 MHz. Despite the short specimen length, event location was applied mainly to be able to separate events occurring within the gauge length and outside.

### 2.3. Thermography

Infrared (IR) thermography was applied on the other side of the specimen to monitor the temperature variation caused by cyclic loading. The experimental setup included an IR midwavelength camera (CEDIP, 3–5 *μ*m) with a cooled indium antimonide (InSb) detector and a focal plane array (FPA) with pixel format of 320 (H) × 240 (V) and sensitivity of 20 mK. In order to achieve the best field of view (FOV), the IR camera was mounted 30 cm in front of the specimen [33, 34]. The surface temperature was recorded with a sampling rate of 100 Hz.

## 3. Results

### 3.1. Calm Ratio

**Figure 3:**
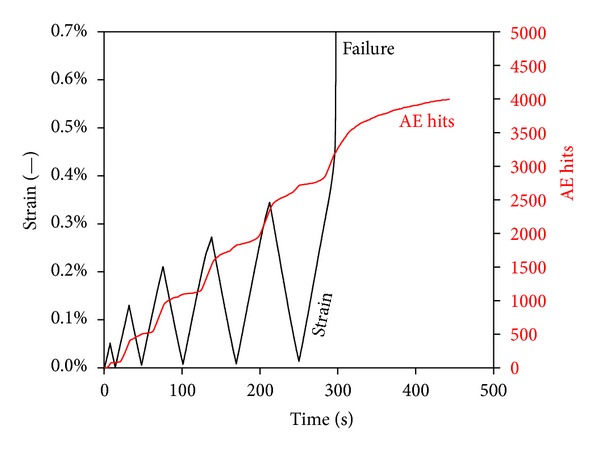
Strain and AE cumulative history for specimen B.

**Figure 4:**
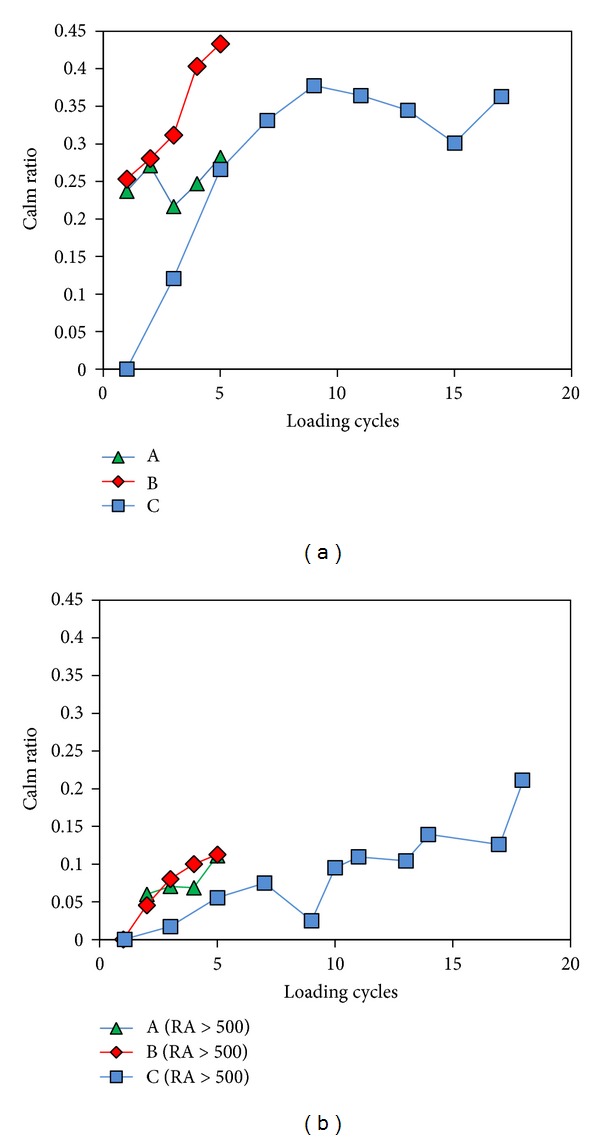
Calm ratio for specimens (A, B, and C) (a) total activity and (b) hits with RA > 500 **μ**s/V.

Figure 3 indicatively shows the AE history (cumulative AE signals) along with the strain history for specimen B (notch-to-width 0.35). AE activity builds up to a number of approximately 4000 hits. However, as expected, the rate of acquisition is not constant but exhibits fluctuations according to the part of the extension cycle. When load approaches the maximum value of each cycle, AE is recorded in large numbers, as can be seen by the slope of the curve. When the load or strain starts to decrease after the peak of the cycle, the activity though reduced, is not actually diminished to zero. The AE activity during the unloading stage over the total AE activity of each cycle is called “Calm ratio, CR.” It has been seen that, as damage is being accumulated in the material, activity during unloading increases [7, 8]. This is the result of irreversible damage that complicates stress relief when external loading reduces in multiphase materials. Therefore, values of CR near zero indicate good structural health, while increase of calm ratio is connected to accumulated damage.  Figure 4(a) shows the development of CR for specimens with different notches subjected to the loading protocol. For specimens A and B, CR starts from quite high value of 0.25. For specimen B it continuously increases to the value of 0.43 until the last full cycle (fifth, while macroscopic fracture occurred within the sixth). This value denotes that the activity during unloading is almost half of the one during loading implying heavily deteriorated stage of material according to the literature in similar fields [7, 8]. For specimen A, CR increases to 0.28 without exhibiting monotonic trend. For specimen C (notch-free), which was tested in a protocol including more frequent cycling, CR starts from the value of zero (more likely because the first cycle was of very low maximum strain producing a small population of AE hits and damage). CR rises monotonically to the level of 0.37 during cycle 9 exhibiting some fluctuations until final failure. It is shown that the trend of the specific indicator follows damage accumulation up to some values which according to the literature denote extensive damage (i.e., 0.35 or more). However, initial CR values of more than 0.2 (seen for two of the specimens) are not expected since the maximum strain in the initial cycle is not expected to impose serious damage. In order to improve the results of the well-established indices in the present case, a procedure to filter out the less significant signals based on AE waveform parameters is explained and proposed below.

**Figure 5:**
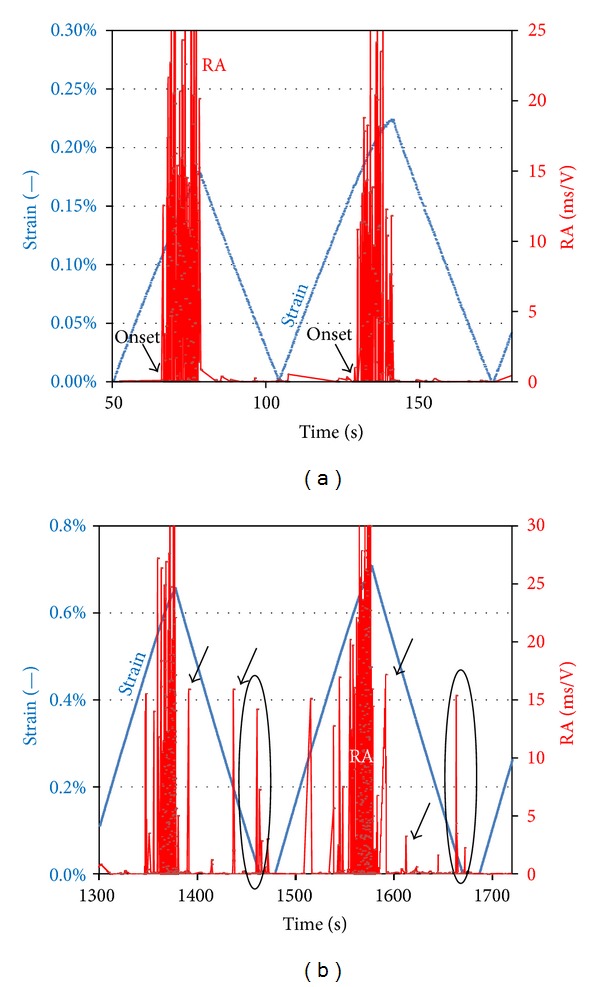
Extension history and RA values for specimen C: (a) 3rd and 4th cycles and (b) 12th and 13th cycles.

As mentioned above, CR depends on the total activity during the different stages of loading and unloading. However, all the signals are not similar; the recorded waveforms exhibit strong qualitative discrepancies and their shape reflects the severity of the source crack. One of the indicators that have been used is the RA, as defined in the introduction. Increase of RA shows an increase of the severity of the events, in certain cases due to the shift from matrix cracking to delaminations or debonding.  Figure 5(a) shows the RA values exhibited during the 3rd and 4th cycles for specimen C. During the increasing phase of the strain, AE hits exhibit quite high RA values of the order of 10 ms/V or more. However, immediately after the peak of the extension cycle and during unloading, the RA of AE hits returns almost instantly to zero level. Therefore, any hit with high RA during loading indicates progressing damage due to a failure mechanism, while during unloading any of the few hits with RA near zero is associated with a totally different mechanism, more likely of irregular stress relief or friction between crack faces.  Figure 5(b) shows later cycles (12th and 13th) of the same experiment. In this case, AE signals with high RA are also exhibited at the unloading stage (denoted by arrows) showing increased damage severity. Therefore, in order to focus only on the high intensity signals, the CR was again calculated for the AE hits with RA > 500 *μ*s/V, in order to exclude signals revealing limited or no fracture intensity. The exact value of 500 *μ*s/V was tentatively selected just in order to exclude signals with negligible RA. The results are shown in Figure 4(b). It is seen that for all specimens CR starts at the level of zero for the first cycle, while it gradually increases to values above 0.1 for specimens A and B, and above 0.2 for specimen C. These results are more reasonable than those of Figure 4(a), since for the first loading cycle, the strain is too low to induce remarkable damage, and therefore the value of CR should be zero or close to zero. With increase of the maximum strain, CR attains values higher than 0.1 which, as reported in the literature, are associated with extensive damage. Additionally, when excluding the low-RA signals the trend is nearly monotonic firmly describing damage accumulation up to failure, while when no filtering is applied (Figure 4(a)), this index seems to attain a saturated value and lose its sensitivity after a number of cycles although damage continues to be accumulated up to failure.

### 3.2. Felicity Ratio

**Figure 6:**
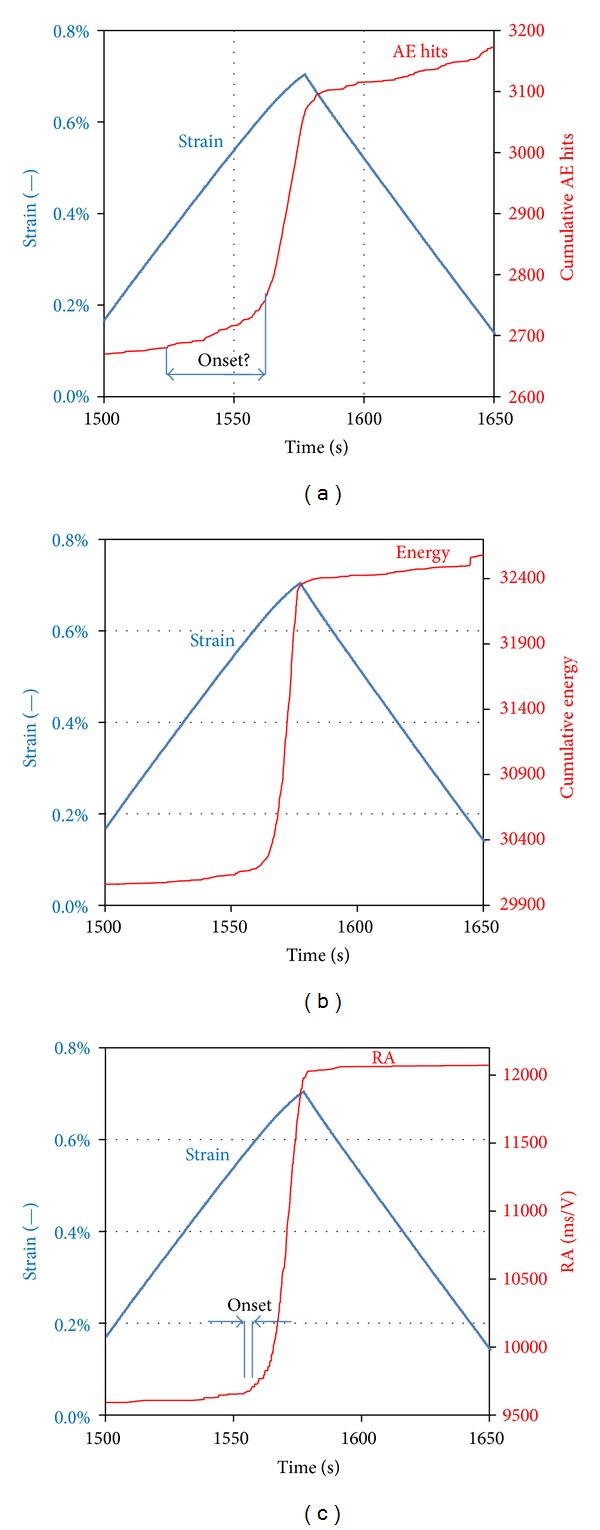
Strain and cumulative (a) AE hit, (b) AE energy, and (c) RA values for the 15th cycle of specimen C.

**Figure 7:**
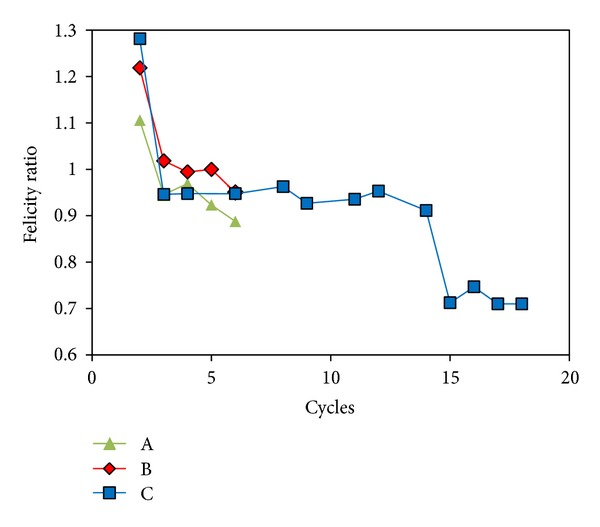
Felicity ratio for different specimens.

Another important index, and one of the first to be applied in AE studies, is the felicity ratio, FR. It is based on the fact that when a material is stressed, this will not result in any emission if the same level of stress has been sustained by the material previously. This is reasonably connected to the cracking procedure since the amount of cracking corresponding to a specific loading level is once created; unloading and reloading upto that point will not cause any more damage. Damage will start accumulating again when the previous stress level has been exceeded, also escorted by AE recordings. In intact materials the above described “Kaiser effect” is valid. However, for a highly damaged material, AE may start earlier than the previous maximum load [1, 8, 12]. The ratio of the load (strain) at which AE is firstly recorded within a cycle over the maximum strain of the previous cycle is called felicity ratio, FR. In order to measure this parameter a clear onset of the AE activity should be targeted for each cycle.  Figure 6(a) shows an example of cumulative hit line along with the strain history focused on a specific loading cycle. AE is continuously recorded and it would be difficult to pick a specific moment as the onset of AE for the specific cycle. Though the rate of incoming activity increases as strain approaches its maximum value, the change is quite smooth making the onset picking troublesome or even impossible. This ambiguity does not allow for reliable evaluation of the onset and hence the FR for the material. Based on the intensity of each signal, Figures 6(b) and 6(c) depict the cumulative energy and cumulative RA for the same specimen and cycle. It is seen that the onset can be much more reliably evaluated and the calculation of FR is enabled without serious ambiguity. This was also seen in Figure 5(a), where only hits with negligible RA are recorded for low strain, while hits of high RA start to emerge only when strain approaches the maximum of each cycle.  Figure 7 shows FR for all specimens as measured by the sharp change in the RA curve. All specimens start with a high FR (above unity), indicating a good material condition at the 2nd cycle. For the next cycles FR decreases to values lower than 1 until reaching the final values of 0.88 and 0.95 for specimens A and B, respectively. Concerning specimen C (unnotched), FR stays approximately constant from the 3rd to the 14th cycle at values around 0.95. At the 15th cycle, FR exhibits a strong drop to 0.7 which is maintained until the final cycle of macroscopic failure. This considerable decrease compared to the initial value indicates a certain deterioration of the material relatively to the initial virgin stage. Values less than 0.95 and especially near 0.7 are connected to quite deteriorated conditions [8, 11, 12]. It is seen that traditional AE indices can be used for damage characterization in this material, while their characterization accuracy is improved when they are enhanced by criteria based on contemporary AE features.

### 3.3. Acoustic Signatures of Positive-Negative Strain

**Figure 8:**
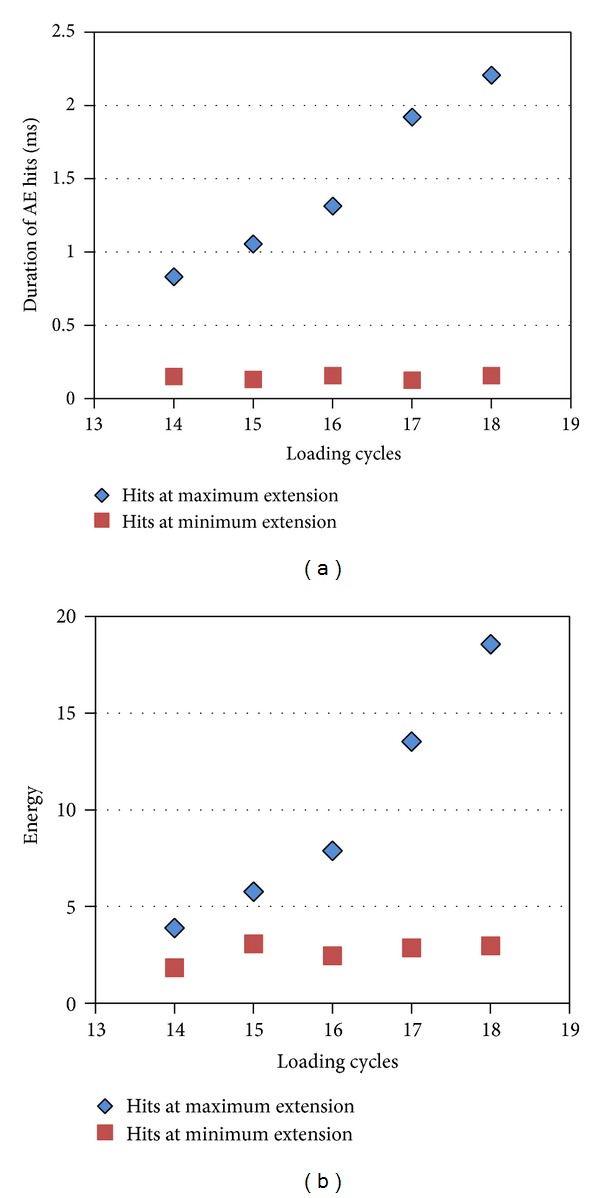
Average value of acoustic emission (a) duration and (b) energy at a time window of 6 s around the maxima and minima of the strain cycles for specimen C.

The sensitivity of AE to monitor different damage mechanisms can also be seen by studying specific experimental parameters in relation to the strain history. It is reminded that the loading protocol was based on extension cycles as macroscopically measured by the testing frame. Strain was also measured externally, by means of a clip gauge, within the central 25 mm of the material. It was observed that, despite the fact that each unloading stage returned to zero load macroscopically, AE activity was recorded in large numbers between the end of each cycle and start of the next, at those instances of otherwise nominally “zero” load conditions (see again the ellipses in Figure 5(b)). This implies that actually the specimen was not strain-free at those moments but under negative strain. This may be connected to the thermal residual stresses related to the manufacturing of ceramic matrix composites [22, 23], while friction between closing crack faces is also a possibility. Thus, the characteristics of AE should be different between sources activated at the positive peak of the strain cycle and the negative peak where the material is under negative strain. Indicatively, the strain curve of Figure 5(a) that concerns early loading cycles does not include negative strain and hence the RA curve in the same figure does not include important AE signals at the minima of the cycles. On the other hand, in Figure 5(b), where strain approaches zero and enters the negative field (at around 1470 s and 1680 s), hits with substantial RA are exhibited at those moments (denoted by ellipses). Separating AE at the maximum and minimum of the cycles shows quite distinct trends concerning certain features, which are dependent on the actual source mechanisms.  Figure 8(a) shows the duration of the AE signals at the maximum and minimum of the cycles (±3 s from positive or negative peaks of strain) for the last five cycles of the testing of specimen C (unnotched). In those cycles, strain clearly deepens into negative values between cycles, as shown in Figure 5(b), and notable AE is recorded at those instances. It is evident that with increasing number of cycles at maximum strain, the duration of the signals increases linearly until the failure of the specimen. This is characteristic of higher intensity fracturing events that occur as the material approaches final failure. However, the AE duration at the return to zero strain is almost constant for any cycle and certainly lower than the tensile side. Similar trends are seen by the energy of the signals in Figure 8(b). For the peak strain of each cycle, AE energy continuously increases, while for the minima, energy remains constant and of lower level. The above comparisons show clearly that AE hits at tensile strain have distinct characteristics from those recorded during negative strain and highlight the sensitivity of AE in recognizing different fracture mechanisms. It can be argued that as tensile load increases, fracture is successively dominated by: matrix crack formation and propagation, interfacial damage and sliding of intact fibers' surfaces across the debonded interface, bridging by single fibers and fiber bundles and fiber pull-out [35]. These mechanisms are known to demonstrate distinct acoustic signatures for a wide range of different materials [12, 17] and therefore an increase in load during successive cycles will cause a continuous increase in the values of these AE indices. On the other hand when strain attains zero or negative values, the characteristics of AE are totally different due to negative strain mechanisms connected to microbuckling phenomena or friction between the crack faces [22].

### 3.4. Real Time Trends

**Figure 9:**
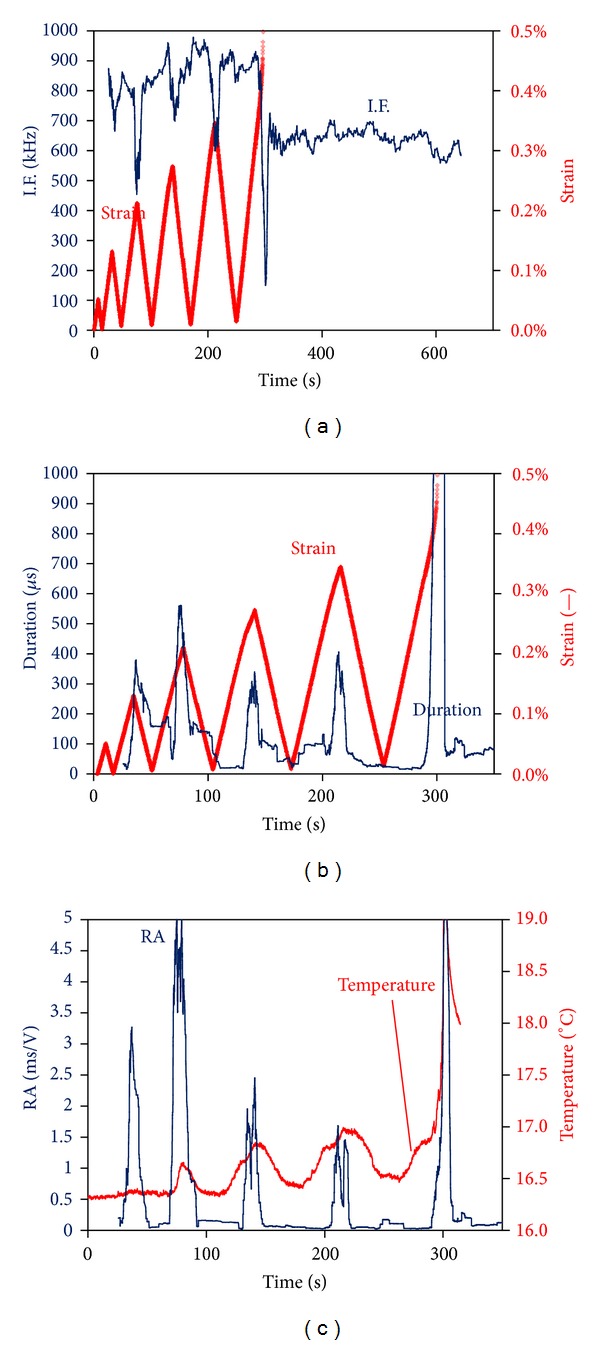
Initiation frequency and strain versus time (a), duration and strain versus time (b), and RA and temperature versus time (c) for specimen B (notch-to-width 0.35).

**Figure 10:**
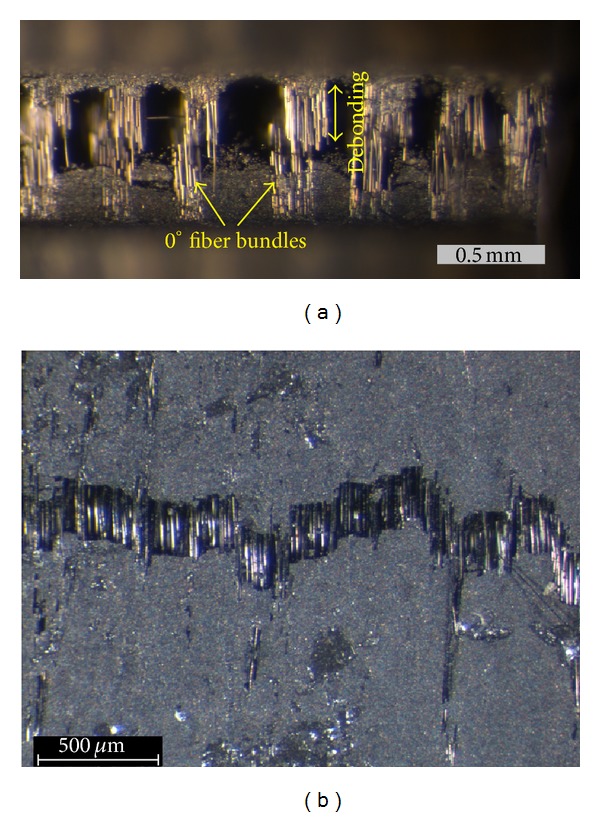
(a) Photograph at the notch of a ruptured specimen. (b) Front view of the crack with continuous fibers bridging the crack sides.

As mentioned in the introduction, different damage mechanisms dominate the material's fracture sequence at different load levels. Since each damage mechanism is related to different AE signatures, it is expected that continuous monitoring of AE will reveal fluctuations based on the loading within each cycle.  Figure 9(a) shows the initiation frequency, I.F., versus time along with the strain history for specimen B. It can be seen that between the peaks of each extension cycle, I.F. is at high levels of approximately 800 kHz, while at peak strains, when the macroscopically maximum tension occurs, I.F exhibits local minima. It is noteworthy that the largest drop is exhibited at the moment of macroscopical failure, reaching values near 200 kHz. Therefore, it is implied that moderate damage occurring at smaller strains can be related to the I.F. value of 800 kHz, while macrofracturing events with I.F. of 200 kHz. It is mentioned that the I.F. line is the moving average of the recent 100 hits. For the specific specimen, the acquisition of AE activity was not halted at macroscopic failure as AE events were still being recorded with a high rate (see also Figure 3). At the moment of fracture (approx. at 300 s) a visible crack was developed from one notch to the other. However, fibers were still bridging the crack, enabling removal of the specimen in one piece after the end of the experiment. This shows that a part of the fibers' population did not fail at the crack opening but preferably within the matrix environment. The continuous AE activity after load drop can only be discussed in terms of failed fiber sliding (pull-out) through the matrix, since the rest of the specimen is almost load free. It is quite interesting to note that after specimen failure and while only pull-out could be active among all damage mechanisms, I.F. is restored to approximately 600 kHz which is higher than the I.F. at main fracture but certainly lower than 800-900 kHz corresponding to matrix cracking at low loads. This AE behavior during pull-out is similar to the one of steel fiber reinforced concrete (SFRC) under bending [17] with fiber pull-out mechanism exhibiting frequency characteristics that are lower than tensile matrix cracking.  Figure 9(b) shows the trend of AE duration for the same specimen for the first 300 s, until failure. Similarly to I.F., duration also exhibits fluctuations with load, but in this case it increases at the points of maximum strain (at approximately 300 *μ*s, much higher than its level at low strains, less than 100 *μ*s). As discussed earlier, this could be the effect of increasing proportion of interfacial debonding and sliding of intact fibers across the debonded interface with the matrix that is reasonable to occur at the higher strains of each cycle. Visual evidence of sliding between fiber bundles and off-axis layers can be seen in the microphotograph of Figure 10(a) which is the postmortem side view of a specimen's notched ligament. The crack opening is of the order of 500 *μ*m, while debonding between fiber bundles and the off-axis plies is of similar length; hence, also the corresponding pull-out length scale between fibers bundles and the matrix. It is concluded that although rupture of numerous fibers is visible in the figure, the bundles did not completely break, see also Figure 10(b), where continuous fibers are bridging the crack sides. The dominant matrix macrocrack may well have formed during early loading cycles due to the low strength of the off-axis plies, but the final debonding and crack opening occurred at the moment of ultimate failure, resulting also in the explosion of AE duration and drop of I.F. due to the shear characteristics of this mechanism. 

Apart from the insight on the fracture process and active mechanism, the importance lies also on the phenomenological correlations of nondestructively measured parameters with load. Apart from I.F. and duration, Figure 9(c) shows the trend of RA value which again exhibits sharp peaks near the maximum strain of each cycle and is a strong indication of the serious damage mechanisms that are activated at that point. This way, the moments when the material is subjected to high stresses can be easily highlighted just by monitoring AE parameters like duration, RA or I.F., which can be of paramount importance in field applications, where other mechanical measurements may not be applied. For the specific experiment, as mentioned earlier, a thermo-camera was also used to monitor thermal changes throughout the experiment duration. Figure 9(c) shows the maximum temperature of an area of 4 × 12 mm near the left notch of specimen B. These local maxima of temperature correspond to the maxima of strain. From the thermography aspect, this increase is attributed to heating of the specimen owing to high stresses or to microcracking and friction in between crack faces. When strain starts to descent, the temperature gradually decreases because of smooth heat dissipation. The complementary use of thermography indicates these local temperature peaks that coincide with peaks in the RA, which also occur at high tensile strain. This is reasonable since increased stresses result in higher temperature in materials, while at the same time, the same stresses are responsible for fracture phenomena in the microscale that give rise to AE signals with significant severity (high RA and duration and low frequency). Combination of techniques offers the possibility to benchmark the results and give insight in the fracture process of this complex material.

## 4. Conclusions

The present study discusses the acoustic emission behavior of ceramic matrix composites under tensile loading. The coupons were subjected in cyclic loading and different AE parameters were monitored in relation to damage accumulation. AE sheds light into the complicated fracture processes that take place within the specific material. AE indices usually applied in cementitious materials and composites show the potential to characterize damage also in thin coupons of laminated ceramic matrix composites. Calm ratio and Felicity ratio are good indicators of damage, showing monotonic shift with damage accumulation, while in this study their behavior is improved by filtering out signals of limited importance based on their RA value. Specific AE characteristics like energy and duration can be used for identification of different damage mechanisms, since incidents owing to high tensile strain exhibit distinct characteristics from those triggered by negative strain. Online monitoring can also highlight the moments of severe stress of the material, since parameters like initiation frequency, signal duration, and RA exhibit positive or negative peaks at the moments of maximum strain. Additionally, sliding debonding of fiber bundles emits signals of different frequency compared to matrix cracking. The insight given by AE would be difficult to obtain by another type of monitoring method. The study should continue in the direction of standardization of the results, since the AE results heavily depend on the specimen's size and the sensors' response, while combination with other monitoring techniques can further verify the trends.
